# Mesoporous ZnAl_2_Si_10_O_24_ nanofertilizers enable high yield of *Oryza sativa L.*

**DOI:** 10.1038/s41598-020-67611-4

**Published:** 2020-07-02

**Authors:** Fizza Naseem, Yang Zhi, Muhammad Akhyar Farrukh, Fayyaz Hussain, Zongyou Yin

**Affiliations:** 1 Nano-Chemistry Lab, Department of Chemistry, Government College University, Lahore, 54000 Pakistan; 2 Research School of Chemistry, Australian National University, Canberra, ACT 2601 Australia; 3 School of Pharmaceutical Engineering, Shenyang Pharmaceutical University, Shenyang, 110016 China; 4 Department of Chemistry, Forman Christian College (A Chartered University), Lahore, 54600 Pakistan; 5 Land Resource Research Institute, National Agricultural Research Centre, Park road, Islamabad, 44000 Pakistan

**Keywords:** Plant sciences, Environmental sciences, Chemistry, Nanoscience and technology

## Abstract

Controllable release of nutrients in soil can overcome the environmental problems associated with conventional fertilizer. Here we synthesized mesoporous nanocomposite of Zinc aluminosilicate (ZnAl_2_Si_10_O_24_) via co-precipitation method. *Oryza sativa L.* husk was used as source of silica for making the synthesis process green and economical. The nanocomposite was subsequently loaded with urea to achieve the demand of simultaneous and slow delivery of both zinc and urea. The structural characterization of nanocomposite was done by FTIR, XRD, TGA, BET, SEM/EDX and TEM. The release of urea and zinc was investigated with UV–Vis spectrophotometry and atomic absorption spectroscopy, respectively, up to 14 days. It was noted that urea holding capacity of mesoporous ZnAl_2_Si_10_O_24_ nanocomposite over long period of time was increased as compared to bulk aluminosilicates, due to its high surface area (193.07 m^2^ g^−1^) and small particle size of (64 nm). Urea release was found highest in first 24 h because of excess of adsorption on nanocomposite and least at 14th day. Fertilizer efficiency was checked on *Oryza sativa L.* in comparison with commercial urea and results showed significantly higher yield in case of urea loaded ZnAl_2_Si_10_O_24_ nanocomposite.

## Introduction

Increase in soil fertility is the key factor to improve the sustainable crop productivity. Balanced nutrient supply is therefore essential to ensure high crop yield. Nitrogen is the major macronutrients as it is the part of chlorophyll, which is responsible for photosynthesis. It plays a major role in improving the crop yield. Likewise, Zinc is considered the most yield-limiting micronutrient in most plants. Plants fail to develop normally when they are deficient in zinc and nitrogen^1–3^. In 2015 the world’s Nitrogen consumption was about 104.1 million tons and annual average growth was 0.015 million tons^4,5^. However, commercial nitrogen fertilizers are readily soluble in water and release all the nutrients at once. Plants utilizes only 30–40% of the released nitrogen while the remaining 70–60% is lost due to leaching and volatilization. Crop yield to nitrogen surplus rate remains low which leads to the leaching of surplus nitrogen and is the main cause of downstream pollution eutrophication and rising the concentration of greenhouse gas. Slow release of such nutrients in soil by the fertilizer can be helpful to overcome above mentioned problems while minimizing the nitrogen losses and increasing the nitrogen utilization efficiency by plants^6–9^.

Aluminosilicates can be the potentially most suitable material to hold both zinc and urea. Since more than a decade, use of similar minerals for agricultural practices and as enhanced efficiency fertilizer has been being closely observed. Mesoporous materials have gained a special position in this regard^10,11^. Aluminosilicates form crystalline three-dimensional structure of cavities, cages, channels and pores at the nano scale. Their natural structure provides the surface area ranging from 8 to 72 m^2^ g^−1^. Such structures can retain water as well as exchange cations without any major structural change and also have high surface area^12,13^. Therefore, mesoporosity can play a major role in this regard which increases surface area amazingly and increases the retention capacity of aluminosilicates for nutrients. Aluminosilicate frameworks consist of tetrahedral framework if TO_4_ where T = Si^4+^ or Al^3+^, and a resultant negative charge comes to tectosilicate framework when Si is replaced by Al and this negative charge is compensated by cations. Extra framework cations which are present in channels and voids of framework are easily available for cation exchange. Natural and synthetic aluminosilicates are being used for improvement in fertilizers to prolong the release of multiple nutrients in comparison to commercial urea, which in turn can control nitrogen losses due to leaching and volatilization^14–16^. Lateef et al. studied the availability of nutrients from zeolite (a type of aluminosilicates) based nanocomposite upto 7 days in water. It showed that zeolites can retain nutrients like NO_3_^1–^, P_2_O_5_, K_2_O, Na_2_O, Zn^2+^, Ca^2+^ and Mg^2+^ upto considerable amount and time and release them to soil and water gradually^17^. Li et al. tested ammonium and potassium occluded zeolite fertilizer to achieve higher spinach yield and higher oxalate content^18^.

Purpose of this work is to synthesize mesoporous Zinc aluminosilicate nanocomposite and urea will be then loaded into nanocomposite for simultaneous and prolonged delivery of zinc and nitrogen to the plants. Mesopores and surface hydroxides of as-synthesized aluminosilicates can delay the release of urea and zinc in soil up to several days. These type of slow release fertilizer can be utilized as environment friendly multiple nutrient source for plants. Positive results of continual release of nutrients can be observed in enhanced yield and nitrogen recovery efficiency (NRE) in *Oryza sativa L.* plants, when compared to common commercial urea.

## Results

### Structural properties of ZnAl_2_Si_10_O_24_ nanocomposite

**Figure 1:**
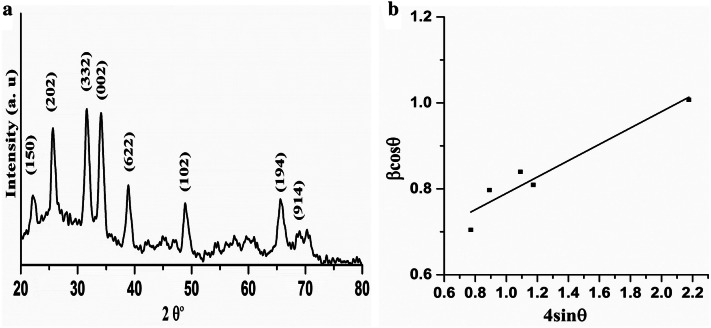
(**a**) X-ray powder diffraction pattern for the ZnAl_2_Si_10_O_24_ nanocomposite (**b**) W–H Plot from XRD pattern of ZnAl_2_Si_10_O_24_ nanocomposite.

ZnAl_2_Si_10_O_24_ nanocomposite was analyzed by X-Ray diffraction technique (XRD, PANalytical Empyrean diffractometer, Netherland). XRD pattern was plotted from 20° to 80° of 2 theta region as shown in Fig. 1a. The characteristic peaks at 22.1°, 25.96°, 31.66°, 38.9°, 65.7°, 68.8° were attributed to Al_2_Si_10_O_24_ for the reflection planes (150), (202), (332), (622), (194), (914), respectively, and are matched with PDF# 00-006-0239 and PDF# 00-029-1257. Peaks at 34.1° and 48.9° for reflection planes (002) and (102) of ZnO, respectively, are accorded to PDF# 00-003-0888^19^. As in our case zinc metal is used so XRD spectra can be attributed to ZnAl_2_Si_10_O_24_.

Crystallite size at reflection plane of 332 was calculated from X-Ray diffraction pattern by using Scherrer Eq. (1) which was estimated approximately 26.9 nm.

1 $$ D = \frac{K\lambda }{{\beta \cos \theta }} $$

where *D* is particle size, *θ* is Bragg’s diffraction angle, *β* is full width at half maximum (FWHM) and *λ* is wavelength of X-ray *λ* = 1.54 Å, *K* is constant with the value 0.89.

Average crystallite size was also calculated through William–Sons Hall Eq. (2).

2 $$ \beta \cos \theta = \frac{K\lambda }{D} + 4\varepsilon \sin \theta $$

where *ε* is strain of crystal. A graph was plotted between $\beta \cos \theta$ and $4\sin \theta$ to have straight line shown in Fig. 1b. From the equation of straight line, intercept was calculated to determine the estimated value of crystallite size and slope to determine the strain. Crystallite size calculated from William-sons hall equation was found 24.4 nm and strain was 1.9 × 10^–1^. Crystallite size calculated from William-sons hall equation is smaller as this equation considers strain and depends upon 1/tanθ while Scherrer equation depends upon 1/cos*θ*^20,21^.

### Fourier transform infrared spectroscopy (FTIR)

**Figure 2:**
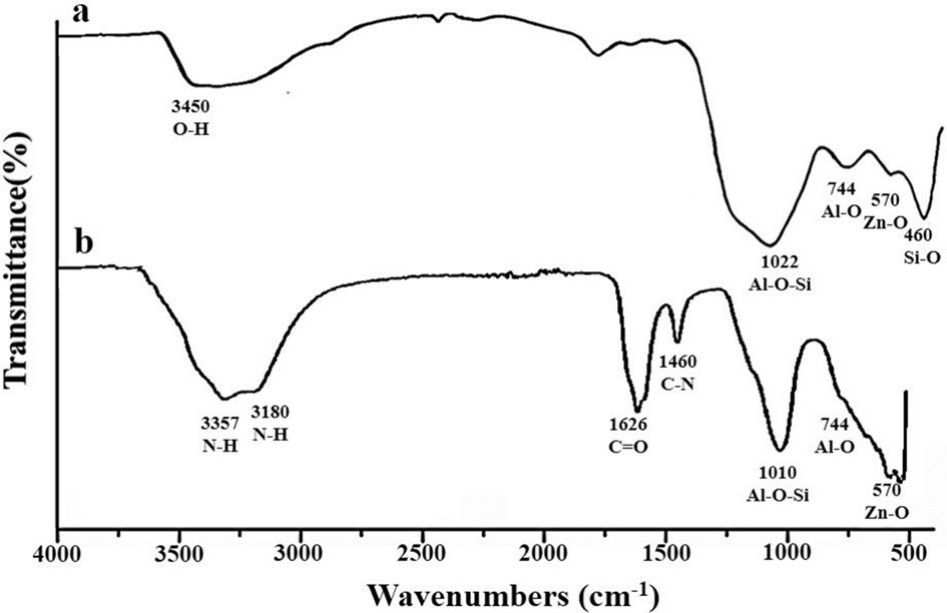
FTIR spectra of (**a**) ZnAl_2_Si_10_O_24_ nanocomposite, (**b**) urea loaded ZnAl_2_Si_10_O_24_ nanocomposite.

FTIR (IR Prestige-21, Shimadzu) was extensively used for the identification of functional groups. Figure 2a exhibits Zinc aluminosilicate having broad transmission band at 3,450 cm^−1^ attributed to OH due to absorbed water molecules and surface silanoles. The broad band at 1,022 cm^−1^ is due to Al–O–Si bridges. Peaks at 744 cm^−1^ and 460 cm^−1^ are due to external tetrahedral vibrations and internal bending of Al/SiO_4_ tetrahedra, respectively. Band at 570 cm^−1^ is attributed to Zn–O^22,23^.

Figure 2b showed the FTIR for the UZAS nanocomposite. A wide band is observed with two peaks at 3,357 cm^−1^ and 3,180 cm^−1^ representing the stretching vibrations of –NH_2._ The peaks at 1626 cm^−1^ and 1,460 cm^−1^ represent stretching vibrations of C=O and C–N bonds^24^. The band at 1,010 cm^−1^ represents Al–O–Si bonds. While Al–O peak at 744 cm^−1^ and Zn–O peak is at 570 cm^−1^_._

### Thermo gravimetric analysis (TGA)

**Figure 3:**
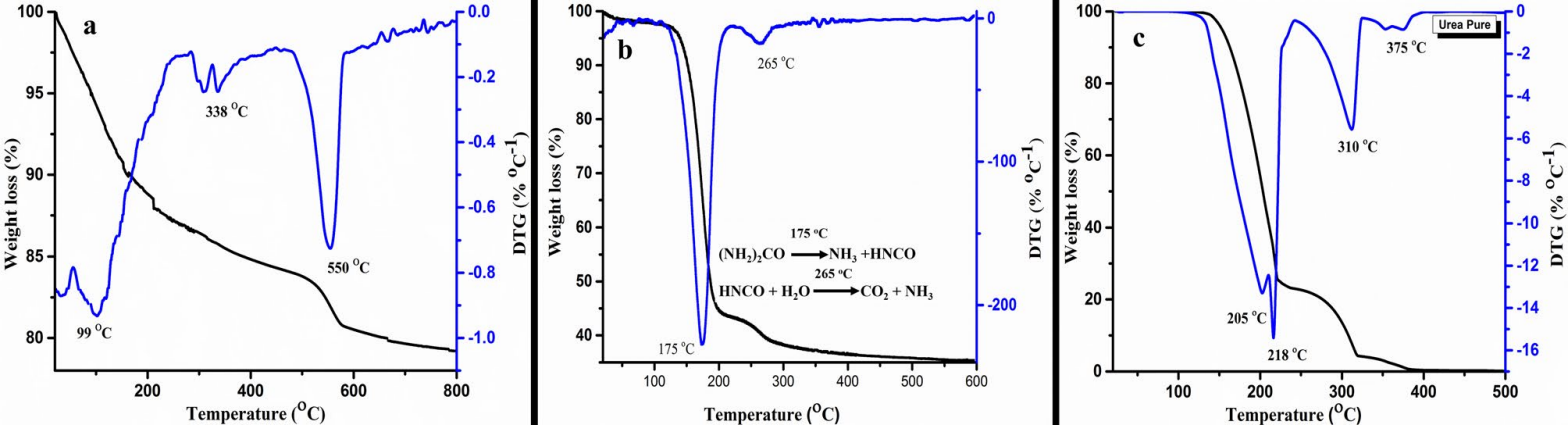
(**a**) TGA and DTG of uncalcined ZnAl_2_Si_10_O_24_ nanocomposite, (**b**) TGA and DTG of Urea loaded nanocomposite (UZAS) (**c**) TGA and DTG of pure urea.

TGA was done by using thermal analyzer TA SDT Q600. Figure 3a shows the thermo gravimetric analysis of uncalcined sample of Zinc aluminosilicate.

Derivative of TGA curve was calculated by using equation as follows in Eq. (3).

3 $$ {\text{DTG}} = {\raise0.7ex\hbox{${\left( {{\text{w}}_{{{\text{t}} + \Delta {\text{t}}}} - {\text{w}}_{{{\text{t}} - \Delta {\text{t}}}} } \right)}$} \!\mathord{\left/ {\vphantom {{\left( {{\text{w}}_{{{\text{t}} + \Delta {\text{t}}}} - {\text{w}}_{{{\text{t}} - \Delta {\text{t}}}} } \right)} {2\Delta {\text{t}}}}}\right.\kern-\nulldelimiterspace} \!\lower0.7ex\hbox{${2\Delta {\text{t}}}$}} $$

where w_t+Δt_ and w_t-Δt_ the remaining weight of sample at time t + Δt and t − Δt, respectively, and Δt is the time interval for between two readings.

Three distinct regions of weight loss are noted also verified by the DTG curve shown in Fig. 3a. First weight loss of 12% at 50–200 °C which corresponds to the loss of physioadsorbed eight water molecules from ZnAl_2_Si_10_O_24_·8H_2_O. Second weight loss of 4.1% in range of 250–450 °C is due to the loss of surfactant template in form of CO_2_. While third 3.5% weight loss at 555 °C is due to the condensation of adjacent silanoles and removal of two water molecules.

Urea loading was also investigated by analyzing the thermal decomposition of urea loaded nano composite. TGA of urea loaded nanocomposite in Fig. 3b shows total weight loss of urea, i.e. 56% of urea, is lost from the nanocomposite. Figure 3c demonstrates pure urea completely decomposes above 380 °C. Decomposition of urea over nanocomposite occurs at lower temperatures and in two major steps, instead of three major steps. This may be attributed to pores and channels of aluminosilictes which inhibits the formation of side products like ammelide, ammeline and melamine formed during pure urea decomposition process^25,26^.

### Particle size analysis

The particle size of ZnAl_2_Si_10_O_24_ nanocomposite was measured by using Dynamic Light Scattering **(**DLS) technique (BT-90 nano particle size analyzer instrument). It measures intensity of light scattered by the movement of particles (Brownian motion) when they are perpendicular to the light source. Stokes–Einstein equation is used to calculate the hydrodynamic diameter of these particles^22^. A stock solution was prepared by adding 2.5 mg of ZnAl_2_Si_10_O_24_ nanocomposite in 10 mL water and ultrasonicated for 2 h to make homogenous colloidal solution. Then 10 µL of stock solution was transferred to a 1 mL cuvette and rest was filled with water. The average particle size (D 4, 2) of 55.2 nm with 51 m^2^ g^−1^ surface area was measured by subjecting diluted solution in particle size analyzer.

### Brunauer–Emmett–Teller (BET)

**Figure 4:**
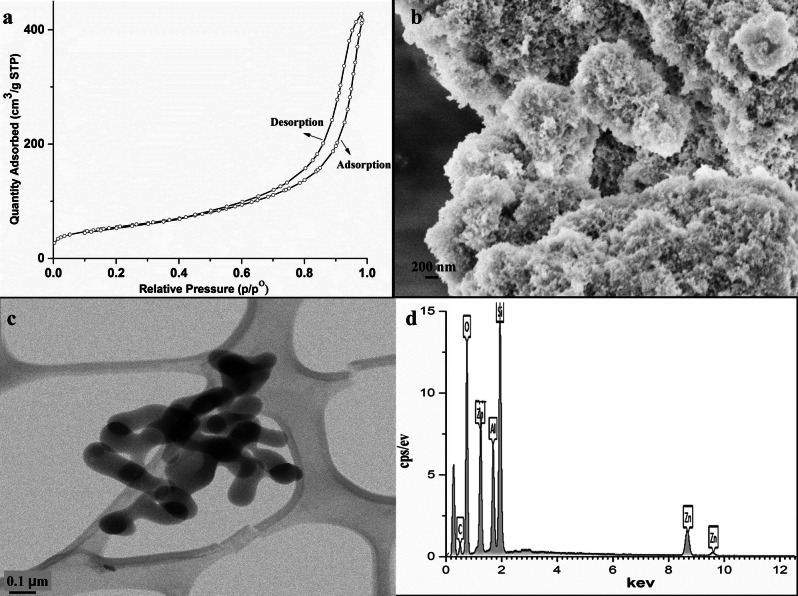
Characterization of ZnAl_2_Si_10_O_24_ nanocomposite, (**a**) BET, N_2_ adsorption desorption isotherm (**b**) SEM (**c**) TEM d) EDX.

Surface area was determined through Brunauer–Emmett–Teller (BET) technique. The initial weight of the sample was 0.0225 g while final weight was 0.0215 g and the degassed temperature was 150 °C. BET surface area was found 193.07 m^2^ g^−1^ while average pore size was 13.17 nm. Increase in BET surface area with respect to particle size analysis may be attributed due to the porosity of the material. As shown in Fig. 4a, BET curve clarifies presence of mesopores in as synthesized nanocomposite through its type IV isotherm and hysteresis loop.

### SEM/EDX

Scanning electron microscopy (SEM, JEOL, JSM-6480) image in Fig. 4b shows the porous surface morphology of the sample. Energy dispersive X-ray (EDX) image in Fig. 4d confirms the presence of weight percentage composition of Si, Al, Zn and O. Table 1 shows the experimental and theoretical values of the elemental composition.

**Table 1:** EDX and theoratical weight percent of ZnAl_2_Si_10_O_24_ nanocomposite.

Element	EDX wt %	Theoretical wt %
Al	8.17	6.8
Si	26.25	35.8
Zn	8.62	8.2
O	51.01	48.7

### Transmission electron microscopy (TEM)

Transmission electron microscope (model JEM-ARM200 F UHR) image (Fig. 4c) shows that the ZnAl_2_Si_10_O_24_ nanocomposites are present in agglomerations but have a distinctive spherical structure with an average particle size of 64 nm. Which is comparable with particle size analysis result.

### Release of urea in water

**Figure 5:**
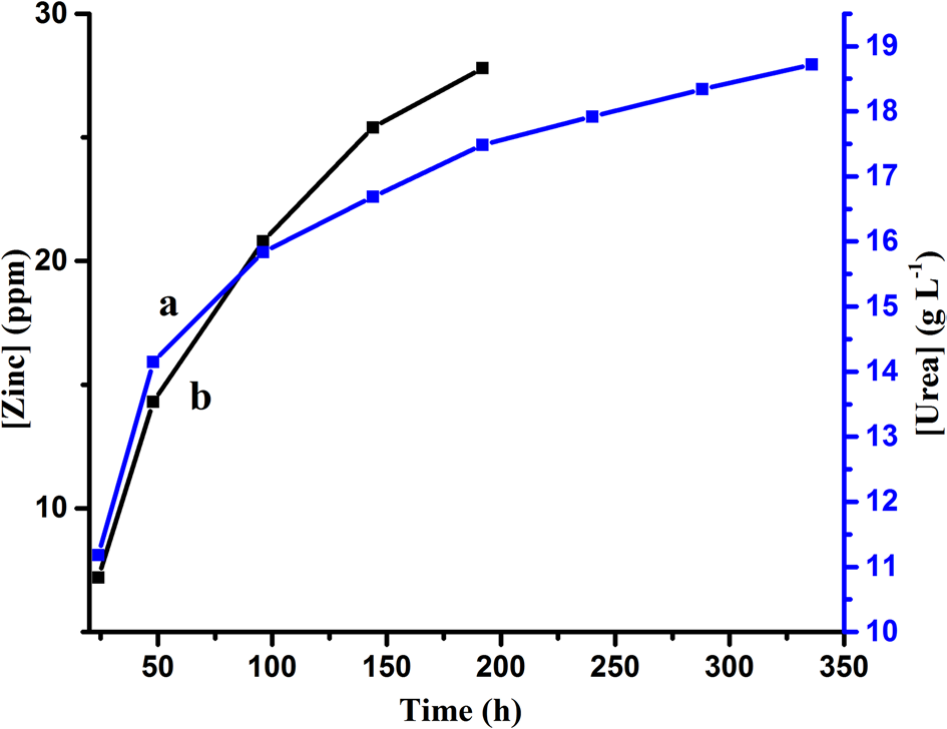
Release of (**a**) urea and (**b**) zinc in water from urea loaded ZnAl_2_Si_10_O_24_ nanocomposite.

Using UV–visible spectroscopy method, it was noted 19.31 g L^−1^ of urea out of 40 g L^−1^ urea solution remained behind in supernatant solution after loading of urea. Which determines 20.69 g L^−1^ was loaded on zinc aluminosilicate nanocomposite. The release of urea from urea loaded ZnAl_2_Si_10_O_24_ nanocomposite in water was observed for 14 days by using UV–visible spectrophotometer. The concentration of released urea in water from ZnAl_2_Si_10_O_24_ nanocomposite was 11.182 g L^−1^ after 24 h. Urea concentration was determined after 48, 96, 144, 192, 240, 288 and 336 h which was 2.964, 1.689, 0.851, 0.800, 0.432, 0.416, 0.380 g L^−1^, respectively. The maximum release of urea was 11.182 g L^−1^ within 24 h due to excess of adsorbtion of urea on nanoparticles and later on a steady release was observed from 4 to 7th day. From 10th day, the concentration of released urea was remained half of the concentration released on 4th day and it started to decline further to 14th day. A graph (Fig. 5) shows cumulative release of urea with time.

### Release of zinc in water

Release of zinc in water from ZnAl_2_Si_10_O_24_ nanocomposites was determined by the atomic absorption spectroscopy. Zinc release was measured at 24, 48, 96, 144 and 192 h which was 7.2, 7.1, 6.5, 4.6 and 2.4 ppm, respectively. The graph of concentration zinc released from ZnAl_2_Si_10_O_24_ nanocomposite against time shows a steady release of zinc ion in water for 3 days. The concentration of released zinc ion gradually decreases with the passage of time and lowest value was found on 10th day. Figure 5 displays the release of zinc with time.

### Characterization of soil and urea loaded zinc aluminosilicate (UZAS)

The pH of soil used for experiment was 7.95 and electrical conductivity was EC (w/v = 1/1) 451 uS/cm. Nitrate nitrogen contents of the soil were 0.50 mg kg^−1^ and phosphorus 1.70 mg kg^−1^ and zinc 0.8 mg kg^−1^. pH of urea was 7.41, pH of UZAS was 7.92. Percent total nitrogen in urea was 44% and total nitrogen of UZAS was 23%.

### Comparison of urea and UZAS fertilization on *Oryza sativa L.* yield and nitrogen recovery efficiency (NRE)

Fertilizer treatment significantly effects the productivity of the plants. Table 2 shows amount of nitrogen applied in pots of *Oryza sativa L.* for six different treatments from T1 to T6 for both sources of nitrogen e.g. UZAS and commercial urea. A detail experimental procedure is given in “Pot experiment using *Oryza sativa L.*”. Statistical analysis was done for comparing the effect of both nitrogen sources on *Oryza sativa L.* traits like number of tillers, yield, harvest index (HI) and NRE.

**Table 2:** Rates of nitrogen applied to different treatments.

S. no	Nitrogen applied (mg kg^−1^)	Nitrogen applied (g pot^−1^)
T1	0 (control)	0 (control)
T2	30	0.45
T3	60	0.90
T4	90	1.35
T5	120	1.80
T6	150	2.25

Treatment with control conditions showed the minimum number of tillers for both sources of nitrogen (20.33) and highest number of tillers were observed at highest rate of nitrogen in T6, 50.00 for commercial urea and 56.33 for UZAS as shown in Table 3. While comparing commercial urea with UZAS it was observed that total number of tillers in case of treatments T1, T2 and T4 remain non-significant and results showed significant increase in total number tillers in T3, T5, and T6 where nitrogen is applied at rate of 60, 120 and 150 mg kg^−1^ in case UZAS as source of nitrogen. Numbers of productive tillers were significantly higher in case UZAS in comparison to commercial urea in all treatments except control. Treatment’s mean homogenous result for productive tillers of UZAS was 37.94 while in case of commercial urea mean was 34.89.

**Table 3:** Effect of nitrogen treatments and sources on number of total and productive tillers of *Oryza sativa L.*

N applied (mg kg^−1^)	Number of tillers					
	Commercial urea		Urea loaded ZnAl_2_Si_10_O_24_		Total mean	Productive mean
	Total	Productive	Total	Productive		
0	20.33 ± 2.08^ns^	19.33 ± 1.5^ns^	20.33 ± 0.58^ns^	20.33 ± 0.58^ns^	20.33 F	19.83 F
30	29.33 ± 1.53^ns^	23.67 ± 1.5^s^	26.67 ± 1.53^ns^	26.33 ± 2.08^s^	28.00 E	25.00 E
60	34.33 ± 0.58^s^	33.00 ± 1.0^s^	37.33 ± 1.53^s^	35.33 ± 0.58^s^	35.83 D	34.17 D
90	41.33 ± 2.08^ns^	39.33 ± 0.5^s^	42.00 ± 1.00^ns^	41.67 ± 1.53^s^	41.67 C	40.50 C
120	48.67 ± 2.08^s^	44.00 ± 1.7^s^	51.33 ± 1.15^s^	47.67 ± 1.53^s^	50.00 B	45.83 B
150	52.00 ± 0.58^s^	49.33 ± 1.5^s^	56.67 ± 2.08^s^	56.33 ± 0.58^s^	54.33 A	52.83A
Mean	37.22 **B**	34.89 **B**	39.50 **A**	37.94 **A**		
Productive tillers LSD treatment × source (0.05) = 2.252 Total tillers LSD treatment × source (0.05) = 2.601						

^ns^Nonsignificant difference between commercial urea and urea loaded ZnAl_2_Si_10_O_24_ within same treatment.

^s^Significant difference between commercial urea and urea loaded ZnAl_2_Si_10_O_24_ within same treatment.

Paddy yield difference is significant in treatments T3, T4 and T6 where nitrogen is applied at rate of 60, 90 and 150 mg kg^−1^ and higher in case of UZAS in comparison to commercial urea. Commercial urea showed the mean of treatments for paddy yield 43.57 g while UZAS showed mean yield 46.00 g, there is significant difference in both yields. As given in Table 4, finest results were observed in T5 for both sources of nitrogen 57.27 g for commercial urea and 59.13 g for urea UZAS. However, the value for harvest index (HI), which is ratio of paddy yield to cumulative paddy and straw yield, was found highest in T4 which remains 41.67 for commercial urea and significantly higher for UZAS 43.00.

**Table 4:** Effect of nitrogen treatments and sources of nitrogen on paddy yield of *Oryza sativa L.*

N applied (mg kg^−1^)	Yield (g)					
	Commercial urea			Urea loaded ZnAl_2_Si_10_O_24_		
	Straw	Paddy	HI	Straw	Paddy	HI
0	45.56 ± 3.97^ns^	27.00 ± 1.08^ns^	37.00 ± 1.11^ns^	45.80 ± 4.20^ns^	27.33 ± 1.27^ns^	37.33 ± 1.07^ns^
30	55.36 ± 0.42^ns^	35.06 ± 1.05^ns^	38.67 ± 0.71^ns^	58.73 ± 2.24^ns^	36.86 ± 1.15^ns^	38.67 ± 0.93^ns^
60	61.40 ± 2.87^s^	40.30 ± 1.45^s^	39.33 ± 0.40^ns^	67.33 ± 1.73^s^	44.53 ± 1.86^s^	40.00 ± 1.35^ns^
90	68.33 ± 3.76^ns^	48.63 ± 0.96^s^	41.67 ± 1.09^s^	69.80 ± 1.78^ns^	52.10 ± 1.23^s^	43.00 ± 0.26^s^
120	80.67 ± 1.91^ns^	57.27 ± 1.76^ns^	41.33 ± 0.41^s^	82.77 ± 2.05^ns^	59.13 ± 2.05^ns^	41.67 ± 0.64^s^
150	82.80 ± 2.58^s^	53.16 ± 0.93^s^	39.00 ± 0.45^ns^	87.97 ± 3.16^s^	55.87 ± 2.35^s^	39.33 ± 0.75^ns^
Mean	65.69 **B**	43.57 **B**	39.61 **A**	68.65 **A**	46.00 **A**	39.94 **A**
	Paddy weight LSD treatment × source (0.05) = 2.460 Straw weight LSD treatment × source (0.05) = 5.940 Harvest index (HI) LSD treatment × source (0.05) = 1.550					

^ns^Nonsignificant difference between commercial urea and urea loaded ZnAl_2_Si_10_O_24_ within same treatment.

^s^Significant difference between commercial urea and urea loaded ZnAl_2_Si_10_O_24_ within same treatment.

Statistical analysis showed there is no prominent difference in the nitrogen percent of the paddy for the T1, T3 and T6 by comparing urea and UZAS. However, there is significant difference of nitrogen percent in paddy for T2, T4 and T5. Table 5 shows the least paddy nitrogen percentage is from the control treatment, while the best results were shown by T5 where total nitrogen was 1.87% in paddy when source was commercial urea and 1.94% when source was UZAS. In T6, straw nitrogen percent was found highest 0.65% where the source was UZAS, while in case of commercial urea it was 0.62%.

**Table 5:** Effect of treatments and sources of nitrogen on percentage total nitrogen of straw and paddy of *Oryza sativa L.*

N applied (mg kg^−1^)	Total nitrogen (%)			
	Commercial urea		Urea loaded ZnAl_2_Si_10_O_24_	
	Straw	Paddy	Straw	Paddy
0	0.43 ± 0.04^ns^	1.20 ± 0.04^ns^	0.43 ± 0.01^ns^	1.27 ± 0.02^ns^
30	0.45 ± 0.04^ns^	1.49 ± 0.02^s^	0.47 ± 0.03^ns^	1.35 ± 0.01^s^
60	0.61 ± 0.02^s^	1.65 ± 0.01^ns^	0.49 ± 0.01^s^	1.71 ± 0.03^ns^
90	0.67 ± 0.08^s^	1.66 ± 0.01^s^	0.56 ± 0.04^s^	1.93 ± 0.05^s^
120	0.58 ± 0.05^s^	1.87 ± 0.11^s^	0.53 ± 0.02^s^	1.94 ± 0.04^s^
150	0.62 ± 0.03^ns^	1.80 ± 0.03^ns^	0.65 ± 0.09^ns^	1.84 ± 0.09^ns^
Mean	0.57 **A**	1.61**A**	0.50 **B**	1.67 **A**
CVC paddy total nitrogen (%) LSD treatment × source (0.05) = 0.0883 CVC straw total nitrogen (%) LSD treatment × source (0.05) = 0.0761				

^ns^Nonsignificant difference between commercial urea and urea loaded ZnAl_2_Si_10_O_24_ within same treatment.

^s^Significant difference between commercial urea and urea loaded ZnAl_2_Si_10_O_24_ within same treatment.

Straw nitrogen uptake was highest for the nitrogen rate 150 mg kg^−1^ (0.51 g for commercial urea and 0.58 g for UZAS). While paddy nitrogen uptake is highest with nitrogen applied at 120 mg kg^−1^ (1.07 g for commercial urea and 1.15 g for UZAS). Total nitrogen uptake of paddy was also increased in case of UZAS in comparison to commercial urea as shown in Table 6. Straw nitrogen uptake and total nitrogen of T3, T4 and T5 remain higher for commercial urea, however, paddy nitrogen uptake remains higher for the same treatments of UZAS. This shows the timed delivery of nitrogen from UZAS contributes more towards the paddy growth than straw or vegetative growth.

**Table 6:** Effect of nitrogen treatments and sources of nitrogen on nitrogen uptake of straw and paddy of *Oryza sativa L.*

N applied (mg kg^−1^)	Nitrogen uptake (g)							
	Commercial urea				Urea loaded ZnAl_2_Si_10_O_24_			
	Straw	Paddy	Total		Straw		Paddy	Total
0	0.19 ± 0.03^ns^	0.33 ± 0.00^ns^	0.52 ± 0.03^ns^	0.19 ± 0.02^ns^		0.35 ± 0.02^ns^		0.54 ± 0.04^ns^
30	0.25 ± 0.02^ns^	0.50 ± 0.01^ns^	0.78 ± 0.01^ns^	0.27 ± 0.03^ns^		0.53 ± 0.02^ns^		0.77 ± 0.03^ns^
60	0.38 ± 0.03^ns^	0.66 ± 0.02^s^	1.04 ± 0.05^ns^	0.33 ± 0.02^ns^		0.76 ± 0.03^s^		1.08 ± 0.04^ns^
90	0.46 ± 0.04^s^	0.81 ± 0.08^s^	1.27 ± 0.05^s^	0.39 ± 0.02^s^		1.01 ± 0.04^s^		1.39 ± 0.05^s^
120	0.47 ± 0.04^ns^	1.07 ± 0.05^s^	1.54 ± 0.10^ns^	0.44 ± 0.01^ns^		1.15 ± 0.04^s^		1.58 ± 0.04^ns^
150	0.51 ± 0.04^s^	1.00 ± 0.03^s^	1.47 ± 0.06^s^	0.58 ± 0.11^s^		1.03 ± 0.04^s^		1.60 ± 0.08^s^
Mean	0.378 **A**	0.73 **B**	1.11 **B**	0.37 **A**		0.80 **A**		1.16 **A**
	CVC grain nitrogen uptake LSD treatment × source (0.05) = 0.057 CVC straw nitrogen uptake LSD treatment × source (0.05) = 0.072 CVC total nitrogen uptake LSD treatment × source (0.05) = 0.092							

^ns^Nonsignificant difference between commercial urea and urea loaded ZnAl_2_Si_10_O_24_ within same treatment.

^s^Significant difference between commercial urea and urea loaded ZnAl_2_Si_10_O_24_ within same treatment.

**Figure 6:**
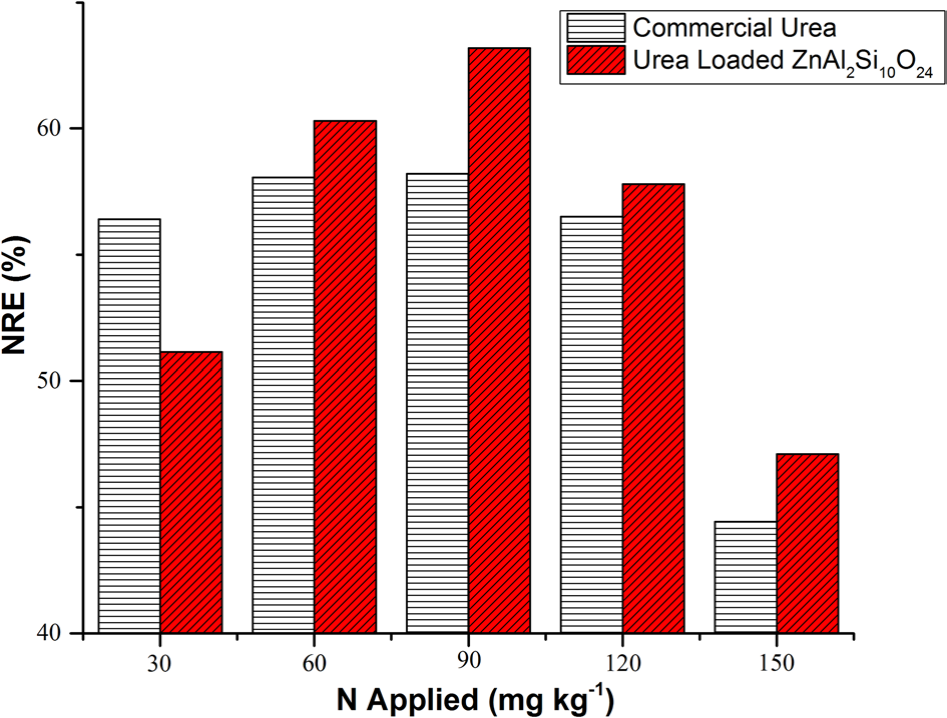
Effect of commercial urea and urea loaded zinc aluminosilicate (UZAS) on Nitrogen recovery efficiency (NRE) of *Oryza sativa L.*

Total nitrogen uptake results shows slow release of urea meaningfully affect the nitrogen recovery efficiency (NRE) of the plants. NRE increases with increased nitrogen applied for both sources till T4 and then decreases as compared to control treatment, shown in Fig. 6. In T2 the NRE for commercial urea is much higher than UZAS and this is because UZAS carry 23% nitrogen which is much lower than commercial urea and does not meet the demand of plant nitrogen at such small level of applied nitrogen due to its slow release property. While in all other treatments the NRE of UZAS is higher than commercial urea meeting the demand of plant. Highest NRE was found for nitrogen applied at 90 mg kg^−1^ in T4 as 63.67% in case of UZAS and 58.19% for commercial urea, as shown in Fig. 6. Vegetative yield is increasing with increase in applied rate of nitrogen and found highest in T6. However, grain yield goes up to maximum in T5 and then decreases in T6, while NRE and HI remains higher in T4. These results indicate that the higher rate of nitrogen applied to T5 and T6 is contributing less towards improvement of plant yield which in turn is the cause of lower NRE and more wastage of urea. However maximum NRE in case of T4 shows most efficient utilization of nitrogen with better yield contents. Zinc concentration in paddy was found in range of 16–18 μg g^−1^ in all treatments and was nonsignficant, as zinc was applied in equal quantity to all treatments. Effect of zinc sulfate and UZAS remain same in all treatments, including those treatments T5 and T6 where zinc sulfate was not added as separate source as demand of zinc was fulfilled by UZAS source itself.

## Discussion

High surface area and porous structure of aluminosilicates make them suitable to hold the urea for longer periods. Surface area of hydrous aluminosilicates ranges from 8 to 72 m^2^ g^−1^ (Clinoptilolite 72.2 m^2^ g^−1^ and Stilbite 8.8 m^2^ g^−1^) and urea holding capacity of clinoptilolite was 120 min^27,28^. High surface area (193.07 m^2^ g^−1^) due to small particle size and mesoporosity (pore size 13 nm determined by BET) of Zinc aluminosilicate nanocomposite enable it to hold urea for longer period of time (336 h) in its pores while surface silanoles makes bonding with urea molecules providing more urea adsorption capacity to nanocomposite. The results obtained for urea loaded on nanocomposite determined by TGA (56% urea loaded) and UV–visible spectroscopy (52% urea loaded) are comparable. Higher value for TGA may be attributed to some extra urea stick with sample. Same dose of nitrogen were applied in case of UZAS and commercial urea to *Oryza sativa L.* plants, however it does affect the productivity of crop and nitrogen recovery efficiency of the plants which is significantly higher in case of UZAS. Vegetative yield increases with higher rate of applied nitrogen but garin yield and harvest index was not dependent on the higher rate of applied nitrogen. Slow delivery of nutrients by UZAS, makes it possible for plants to uptake higher amount of nitrogen for the grain yield. Increase in productivity and NRE is due to slow release property of UZAS as it supplies nitrogen to plant for longer period meeting the demand of plant and results in higher nitrogen uptake. Number of tillers increases with the increase in nitrogen rate for both sources, as nitrogen plays vital role in cell multiplication and tissues formation. This increase in number of tillers leads to increase in vegetative growth. Yield component is not consistent with number of total tillers or productive tillers. Increase in vegetative growth can result into shading which can decrease the grain production by plants. If vegetative growth is greater than requirment, it will lead to lesser paddy yield and will result in lower harvest index. HI is significantly higher in case of UZAS in T4 with nitrogen rate of 90 mg kg^−1^. This is because grain to straw ratio was found highest in that treatment. Slow release property of UZAS leads plants towards more productive tillers and yield in comparison to commercial urea.

Slow release fertilizers increase the nitrogen utilization efficiency of the plants while reducing the environmental damages in comparison to common commercial nitrogen fertilizers. Sulphur coated fertilizers exhibit the common problem of increasing soil pH as both sulphur and urea tend to increase the pH of soil, when applied in large quantities. Similarly polymer coated urea leaves excessive plastic residues in soil. On the other hand, the fertilizer design discussed in this paper is truly green synthesis showing that fertilizer composition such as Zinc aluminum silicate nanocomposite might be most suitable for the soil as it neither harms the pH of soil nor leaves any residues dangerous to the soil. Previous studies show that too much coating applied to urea results in hampering of productive tillers and hence reduction in yield. This was generally attributed to slow initial nutrient supply when plant needed the nutrients in high quantity. Polymer coated urea NRE in rice was found 46% in early rice and 47% in late rice recorded in 2014^29,30^. The nitrogen utilization efficiency of polyolefin coated urea recorded for early rice in 2003 which was 64.0% for nitrogen applied 75 kg ha^−1^ and 27% for the nitrogen applied 150 kg ha^−1^^31^. These results are comparable to results discussed where highest value of NRE was recorded at rate of 90 mg kg^−1^ nitrogen applied for UZAS 63.14% and for commercial urea 58.19% as shown in Fig. 6.

## Materials and methods

All the chemicals were of analytical grade and used without any further purification. *Oryza sativa L.* husk for silica extraction was obtained from Al-Hameed mills, Gujranwala.

### Extraction of silica

*Oryza sativa L.* husk was washed with distilled water and oven dried at 80 °C. Dried husk (15 g) was acid leached by 250 mL of 10% of HCl and refluxed for 2 h. Treated husk was washed with distilled water till pH reaches to 7 and dried in oven at 80 °C. The material was burned in muffle furnace at 700 °C for 2 h to remove incorporated hydrocarbons. 2 g calcined *Oryza sativa L.* husk ash (HA) was again treated with 30 ml of 10% HCl for two hours and washed with distilled water to attain pH 7 and dried overnight at 80 °C. The 0.5 g pretreated husk ash was refluxed for two hours with 20 mL of 1 N NaOH to obtain sodium silicate solution. This solution was allowed to cool at room temperature, filtered with Whatman 41 filter paper and neutralized with 1 N HCl with constant stirring until gel was formed at pH 7. Gel was allowed to age for 18 h then broken by adding water and stirred. Gel was then centrifuged at 6000 rpm for 5 min. The centrifuged material was dried at 80 °C to obtain silica xerogel. The silica obtained was 98.6% pure^32,33^.

### Synthesis of ZnAl_2_Si_10_O_24_

Zinc aluminosilicate ZnO.Al_2_O_3_.Si_10_O_20_ was synthesized through co-precipitation method. For this 1 g of silica as prepared above was added in 20 mL of water then stirred for 5 min, sodium dodecyl sulfate was added at its critical micelle concentration (8 × 10^–3^ mol L^−1^) 48.8 mg along 40 mg of sodium hydroxide with continuous stirring. 5 mL of aqueous 25 mmol aluminium chloride hexa hydrate was prepared and 20 mg of zinc chloride was added to it. The prepared solution was added to silica solution 0.1 ml after each 5 min at feed rate of 0.02 mL min^−1^ with constant stirring for four hours and further stirred for 24 h to get the nanosize. Solution was centrifuged at 12,500 rpm for 2 min and washed with distilled water till pH 7 of filtrate is obtained and material was dried overnight. Dried material was calcined at 550 °C in muffle furnace for 4 h^34^.

### Loading of urea

Urea was loaded on Zinc aluminosilicate. For this purpose, 0.4 g of urea was dissolved in 10 mL of distilled water and 0.2 g of Zinc aluminosilicate nanocomposite was added. The mixture was stirred for 2 h and then centrifuged at 12,000 rpm for 3 min and powder was dried at 60 °C. The unloaded urea in supernatant waste water was investigated by UV–visible spectroscopic method describe in “Particle size analysis”.

### Determination of urea in water

Color reagent was made consisting of p-dimethylaminobenzaldehyde (2 g) dissolved in 100 mL of 95% ethyl alcohol and 10 mL of concentrated hydrochloric acid. Various urea solutions of known concentration in range of 50–400 ppm were made. In urea aliquots 10 mL of above color reagent was added and diluted to 25 mL with distilled water. Absorbance of the yellow green color complex was checked at 420 nm by using UV–visible spectrophotometer (UV-1700 PHARMAspec Shimadzu)^24^. Straight line was obtained by plotting the graph of absorbance against concentration obeying beer lambert law. 0.2 g of UZAS was immersed in 10 ml of water. Solution was filtered after 24, 48, 72, 96, 168, 240 and 336 h and were subjected to UV–visible spectroscopy method to take the respective absorbance values. The value for unknown urea concentration was obtained from by putting the value of absorbance of unknown concentration in graph.

### Determination of zinc in water

Zinc release was determined by using atomic absorption spectrophotometer (AAS) (Shimadzu 88-7000 spectrophotometer). For this purpose standard solution of zinc nitrate of known concentrations of 2, 4, 6, 8 and 10 ppm. Straight line graph was obtained by plotting graph of absorbance against concentration. UZAS 0.2 g was immersed in 10 ml of water. Zinc release in water after 24, 48, 72, 168 and 240 h was determined through AAS by putting the absorbance value of each sample in standard graph and finding out the concentration value.

### Pot experiment using *Oryza sativa L*

A factorial design pot experiment was conducted to check the nitrogen utilization efficiency (NUE) of the plants by using two different nitrogen fertilizers, commercial urea and urea loaded Zinc aluminosilicate (UZAS) as source of nitrogen. Experiment was designed according to 6 different treatments for one source of nitrogen with replication of three. Each pot was filled with 15 kg of soil taken from National Agriculture Research Center (NARC) Islamabad. Cultivar Super was used as test variety of rice. The study was organized with following treatments shown in Table 2. Commercial urea and UZAS were used as nitrogen sources.

First source of nitrogen was commercial urea. In first treatment (T1), urea was not added and consider as controlled while in T2, T3, T4, T5 and T6 20, 40, 60, 80 and 100 mL of 160 mmol urea solution was added and 55.06 mmol solution of zinc sulfate heptahydrate (25 mL/pot) was added.

Second source of nitrogen was UZAS. First treatment was controlled while in T2, T3, T4, T5, T6 20, 40, 60, 80 and 100 mL of UZAS was added. As UZAS is also a source of zinc so lesser quantity of zinc sulfate heptahydrate is required to fulfil the request of zinc. For zinc 92 mL of 25.7 mmol zinc sulfate hepta hydrate was added as in T1, 68 mL in T2, 44 mL in T3 and 20 mL in T4 treatment. In T5 and T6 in which 80 and 100 mL of UZAS was added as source of nitrogen there is no need to add zinc sulfate; as demand is fulfilled by the source itself.

Keeping all other nutrition values constant in each pot, e.g. a mixture of H_3_BO_3_ (38.42 mmol) MgSO_4_·7H_2_O (88.95 mmol), FeSO_4_·7H_2_O (21.49 mmol), CuSO4·5H_2_O (18.88 mmol) was prepared and added 25 mL/pot. 383 mmol solution of potassium sulfate (25 mL/pot). CaHPO_4_·2H_2_O was added in each pot as basal dose of Phosphorus. Fertilizers doses were applied first time before sowing, second time at mid tillering stage and third time at panicles initiation stage. Number of tillers were counted at maximum tillering stage. After harvesting plants paddy was dried for 48 h at 60 °C. Paddy and straw were grounded separately and nitrogen analysis was done by using Kjeldahl method (Kjeldahl analyser, UDK 150). Zinc concentration was determined in paddy by digesting the 0.2 g of grounded sample in 10 ml of mixed acid (HNO_3_ and HClO_4_ 2:1) and heated to 200 °C to obtain clear solution. Solution was diluted to 50 mL and filtered. Zinc concentration was determined through atomic absorption spectrophotometer. Statistics were applied on number of tillers, yield and nitrogen data using statistix 8.1.1.0 via analysis of variance (alpha = 0.05) and means were compared using least significant difference (LSD).

### Soil sampling

Soil samples were collected from NARC Islamabad field in 10–15 cm. Soil was grounded after removal of visible pieces of stones and debris and passed through sieve with mesh size 5. Some soil samples were ground further and passed through 10-mesh or 80-mesh stainless steel sieves for physical and chemical analysis. Selected properties were analyzed as pH (w/v = 1/1), electrical conductivity (EC; w/v = 1/1), texture, soil phosphorus, zinc and nitrate nitrogen.

## Conclusion

The surface area of ZnAl_2_Si_10_O_24_ nanocomposite was calculated 193.07 m^2^ g^−1^ and with urea holding capacity of 20.69 g L^−1^ and retention time increased to about 14 days surpassing the 120 min retention time of clinoptilolite ^28^. While as prepared urea loaded of ZnAl_2_Si_10_O_24_ nanocomposites release the urea up to 336 h in addition with release of zinc which was observed up to 240 h. It was observed that the release of urea was maximum and faster in first 3 days’ while the concentration of zinc released in water was steady as shown in Fig. 6. This prolonged urea release affects significantly on nitrogen utilization efficiency of the *Oryza sativa L.* plants as concluded from statistical analysis. Total nitrogen uptake mean was 1.16 g for the urea loaded Zinc aluminosilicate (UZAS) which was significantly different from the commercial urea where total nitrogen uptake mean was 1.11 g, as given in Table 6. This nitrogen uptake effects the paddy yield data of the plants which is higher in case of urea loaded nanocomposite UZAS as 46.02 g in comparison to commercial urea where mean of treatment for paddy yield is 43.57 g, given in Table 2. Higher nitrogen uptake and higher paddy yield in plants urea loaded nanocomposite in turn effects the NRE of plants which remained higher in urea loaded ZnAl_2_Si_10_O_24_ in comparison to commercial urea. Higher nitrogen recovery efficiency shows that there is less wastage of nitrogen which will lead to less nitrogen leaching in ground water and will be safer for environment.

## Abbreviations

UZAS: Urea loaded zinc aluminosilicate
XRD: X-ray diffraction
FTIR: Fourier transform infrared spectroscopy
TGA: Thermo gravimetric analysis
SEM: Scanning Electron Microscopy
EDX: Energy dispersive X-ray spectroscopy
TEM: Transmission electron microscopy
HI: Harvest index.
